# Protocol for a double-blind randomised controlled trial of low dose intradermal grass pollen immunotherapy versus a histamine control on symptoms and medication use in adults with seasonal allergic rhinitis (PollenLITE)

**DOI:** 10.1186/2045-7022-3-27

**Published:** 2013-08-21

**Authors:** Anna Slovick, Abdel Douiri, Joanna Kelly, Andrea Guerra, Rachel Muir, Konstantinos Tsioulos, Caroline Murphy, Mohamed H Shamji, Sun Ying, Stephen R Durham, Stephen J Till

**Affiliations:** 1 Division of Asthma, Allergy & Lung Biology, King's College London, 5th Floor Tower Wing, Guy's Hospital Campus, London SE1 9RT, UK; 2 Department of Primary Care and Public Health Sciences, King’s College London, 42 Weston St, London SE1 3QD, UK; 3 King’s Clinical Trials Unit, King’s College London, Institute of Psychiatry, 16 De Crespigny Park, London SE5 8AF, UK; 4 Clinical Research Facility, NIHR Biomedical Research Centre, Guy’s Hospital, London SE1 9RT, UK; 5 Section of Allergy and Clinical Immunology, National Heart and Lung Institute, Faculty of Medicine, Imperial College, Dovehouse Street, London SW3 6LY, UK; 6 MRC and Asthma UK Centre for Allergic Mechanisms of Asthma, London SE1 9RT, UK

**Keywords:** Allergy, Vaccine, Clinical Trial, Hayfever, Allergic rhinitis, Randomised, Double-Blind, Placebo, CTIMP, Grass pollen, Intradermal, Injection, Wheal, UKCRC, Clinical Trials Unit, CTU, Clinical Research Facility, CRF, Emergency code break, IMP, Histamine, Timothy Grass, Phleum pratense, Immunology, Immunity

## Abstract

**Background:**

Subcutaneous immunotherapy with high dose grass pollen (typically microgram quantities) was first described over 100 years ago. This treatment suppresses allergen-induced cutaneous late responses, with lesser effects on early responses. We previously reported that repeated 2-weekly intradermal injections of grass pollen - containing approximately 7 ng of major allergen Phl p 5 – led to a progressive suppression of the allergen-induced cutaneous response, and that by the sixth injection, this was inhibited by over 90%. The purpose of this trial is to investigate the clinical efficacy of intradermal desensitisation with low doses (i.e. nanogram quantities) of grass pollen allergen for seasonal allergic rhinitis.

**Methods/design:**

The Pollen Low dose Intradermal therapy Evaluation (PollenLITE) is a single centre double-blind randomised parallel group controlled trial of the efficacy and safety of intradermal grass pollen injections plus standard treatment, versus histamine injections plus standard treatment, in adults with moderate-severe grass pollen-induced allergic rhinitis (‘summer hay fever’). A minimum of ninety adults with a history of moderate-severe persistent allergic rhinitis during the UK grass pollen season will be randomised into two equal groups to receive 7 or 8 intradermal injections of grass pollen extract (containing approximately 7 ng of major allergen Phl p 5) or histamine, before the grass pollen season. In the summer, participants will score their symptoms, medication requirements, visual analogue scores, and complete EuroQOL (EQ-5D-5 L) and mini Rhinoconjunctivitis Quality of Life Questionnaires. Global assessments will also be recorded at the end of the pollen season. Blood samples will be collected from all participants for mechanistic immune assays. Skin punch biopsies will also be collected in 40 participants selected at random from intradermal injection sites after the grass pollen season for mechanistic assays. Finally, to investigate if the desensitising effect of intradermal immunotherapy on cutaneous responses is long-lasting, all participants will be randomised to receive a follow up intradermal injection after 3, 6 or 12 months with measurement of early and late response sizes.

**Discussion:**

Randomisation began in February 2013 and the final participant will complete the trial protocol in August 2014.

**Trial registration:**

ISRCTN 78413121

EudraCT number 2012-002193-31.

## Background

Allergic rhinitis caused by grass pollen affects a quarter of the UK population [1]. Of these, around 5 million people suffer moderate-severe persistent symptoms that have an impact on quality of life, including disturbed sleep, disruption of leisure activities and impairment of performance at work/school [2]. Therefore, there is a substantial unmet need for both therapy and prophylaxis of seasonal allergic rhinitis. In the UK, subcutaneous and sublingual immunotherapy is indicated in patients with moderate-severe symptoms who fail to respond to conventional medications [3]. Immunotherapy i.e. prophylactic inoculation with grass pollen for treatment of season allergic rhinitis was first described in 1911 [4]. The conventional approach involves the regular subcutaneous administration of allergen extracts at high doses (typically microgram quantities of Group 5 grass pollen allergens) [3]. The most commonly used form of grass pollen immunotherapy is that given by injections into the tissue beneath the skin (i.e. subcutaneously) over a period of 2–3 years with increasing amounts of allergen administered weekly for 12 to 15 weeks followed by monthly maintenance injections [3]. A body of evidence, including a Cochrane meta-analysis [5], exists to support the clinical efficacy of high dose subcutaneous immunotherapy. Grass pollen allergen may also be administered at high dose as sublingual tablets or drops, an approach further supported by Cochrane meta-analysis [6]. Both subcutaneous and sublingual high dose immunotherapy have limitations: the vaccine products are expensive and the need for repeated administration in a specialist clinic (subcutaneous immunotherapy) or daily at home (sublingual immunotherapy) is associated with additional expense and/or inconvenience.

‘Proof of concept’ has been established for a novel low dose intradermal desensitisation protocol in subjects with grass pollen-induced allergic rhinitis. A feature of an intradermal allergen injection is the development of local swelling within 6 hours, and persisting for 24–36 hours. This ‘late response’ is characterised by infiltration of inflammatory cells, notably activated TH2 cells, eosinophils and basophils. We showed that six two-weekly intradermal injections of grass pollen containing only 7 ng of major allergen Phl p 5 resulted in a >90% suppression in the cutaneous late response measured after 24 hours in response to these injections [7]. Although the injection sites were alternated between left and right arms, this effect was systemic since responses were also suppressed at a distal site (the back). The magnitude of inhibition was comparable to that seen with a conventional high dose subcutaneous grass pollen vaccine [8] despite equating to over 1000-fold less allergen over the same time period. A grass pollen extract produced by the same manufacturer and given as sublingual immunotherapy delivered 20'000-fold more group 5 allergen over a 10 week period than was given in this study, but was associated with lesser inhibition of late responses, an approximate 40% reduction compared to placebo [9]. We believe that these findings provide a strong rationale for progressing to a clinical trial. The concept of therapeutic intradermal allergen inoculation is not without precedent. In 1926, Phillips, a physician working in Arizona, published a preliminary account of his uncontrolled experiences with intradermal grass pollen immunotherapy in 29 patients [10], extended to 322 patients by 1933 [11], over 90% of whom obtained “satisfactory relief”. However, no randomised controlled trial has yet addressed the efficacy of intradermal low dose allergen injections.

High dose subcutaneous immunotherapy is associated with induction of regulatory T cells (Tregs) [12-14], probably through interaction of CD4+ T cells with pro-tolerogenic dendritic cells (DC). These cells are anti-inflammatory and also induce B cell production of allergen-specific ‘blocking’ IgG antibodies. Low dose intradermal allergen desensitisation is biologically plausible: for example, intradermal injection of radiotracers in animal models results in 100-fold higher rates of drainage to regional lymph nodes than subcutaneous injection [15], potentially leading to more efficient pulsing of lymph node dendritic cells. Also, the dermis is itself an immunologically active environment, rich in Langerhans cells, dermal DC, and lymphatics [16,17]. In contrast, conventional subcutaneous immunotherapy injections target a compartment consisting mostly of connective and adipose tissue but few DC. Irrespective of whether intradermal antigen is processed by DC locally or within draining lymph nodes, we speculate that the basis of the low dose intradermal desensitisation effect is the highly efficient targeting of pro-tolerogenic dendritic cell populations leading to induction of antigen-specific Tregs.

The rationale for the laboratory mechanistic studies using skin biopsies and blood specimens is three-fold. Firstly, there is a need to establish the effects of intradermal immunotherapy on inflammatory cell responses to allergen in tissue (particularly eosinophils, basophils, mast cells and T cells). Suppression of the allergen-induced late response in the skin and nose occurs in parallel following conventional subcutaneous immunotherapy, and the underlying immunological changes within these tissues are extremely similar, if not identical [18-25]. Secondly, we shall investigate if low dose intradermal desensitisation is associated with a systemic immunological effect. We will therefore measure serum allergen-specific IgE and IgG responses and test the biological inhibitory activity of IgG antibodies with an in-house validated assay of IgE-allergen complex binding to B cells [26]. We shall also examine the effect of low dose intradermal allergen treatment on peripheral blood basophil activation in response to grass pollen stimulation ex vivo. There is also a need for exploratory studies to investigate underlying causative mechanisms. These studies will take the form of analysis of T cells expanded from skin biopsy explants in short term cultures. Finally, it is now established that both conventional high dose subcutaneous and sublingual grass pollen immunotherapy exert long-term effects that persist after treatment discontinuation [27,28]. Extended clinical trials involving three successive years of treatment show continued clinical improvement for several years after withdrawal of treatment. To definitively assess such a comparative effect on symptoms/medication use with low dose intradermal immunotherapy would therefore ideally require a prolonged five year trial. Therefore we will perform exploratory studies to investigate the persistence of the intradermal desensitisation effect. We will do this by monitoring how suppression of the skin late response induced by treatment persists over a 12 month follow up period. Therefore the participants in the active and control groups (estimated n = 45 each) will be further sub-randomised to one of three sub-groups (estimated n = 15 each). After having completed their main ‘treatment’ injections, each sub-group will subsequently receive a single follow-up intradermal injection with allergen with late response measurement after 3, 6 or 12 months. For each time point the size of late response in the group that originally received active therapy will be compared with the group that originally received the control intervention.

## Methods/design

We hypothesise that low dose intradermal grass pollen allergen immunotherapy is an effective treatment for seasonal allergic rhinitis (‘hay fever’), reducing symptoms and rescue medication requirements, and improving quality of life for hay fever sufferers.

### Primary objective

To determine if pre-seasonal low dose intradermal grass pollen allergen immunotherapy (either 7 or 8 two-weekly injections of 10 Biological Units (33.3 SQ-U)) reduces symptoms and requirements for anti-allergic drugs in seasonal allergic rhinitis during the 2013 grass pollen season compared to the control intervention (histamine only).

### Secondary objectives

To

1) Determine if this intervention is associated with improvement in quality of life compared to the control intervention, as assessed during the 2013 grass pollen season.

2) Evaluate if this is a safe and well-tolerated form of treatment.

3) Investigate immunological mechanisms associated with this form of treatment, by examining humoral and cellular responses, both in peripheral blood and in tissue.

4) Explore if the intradermal desensitisation effect is long-lived i.e. persists following cessation of intradermal injections.

### Study design

**Figure 1:**
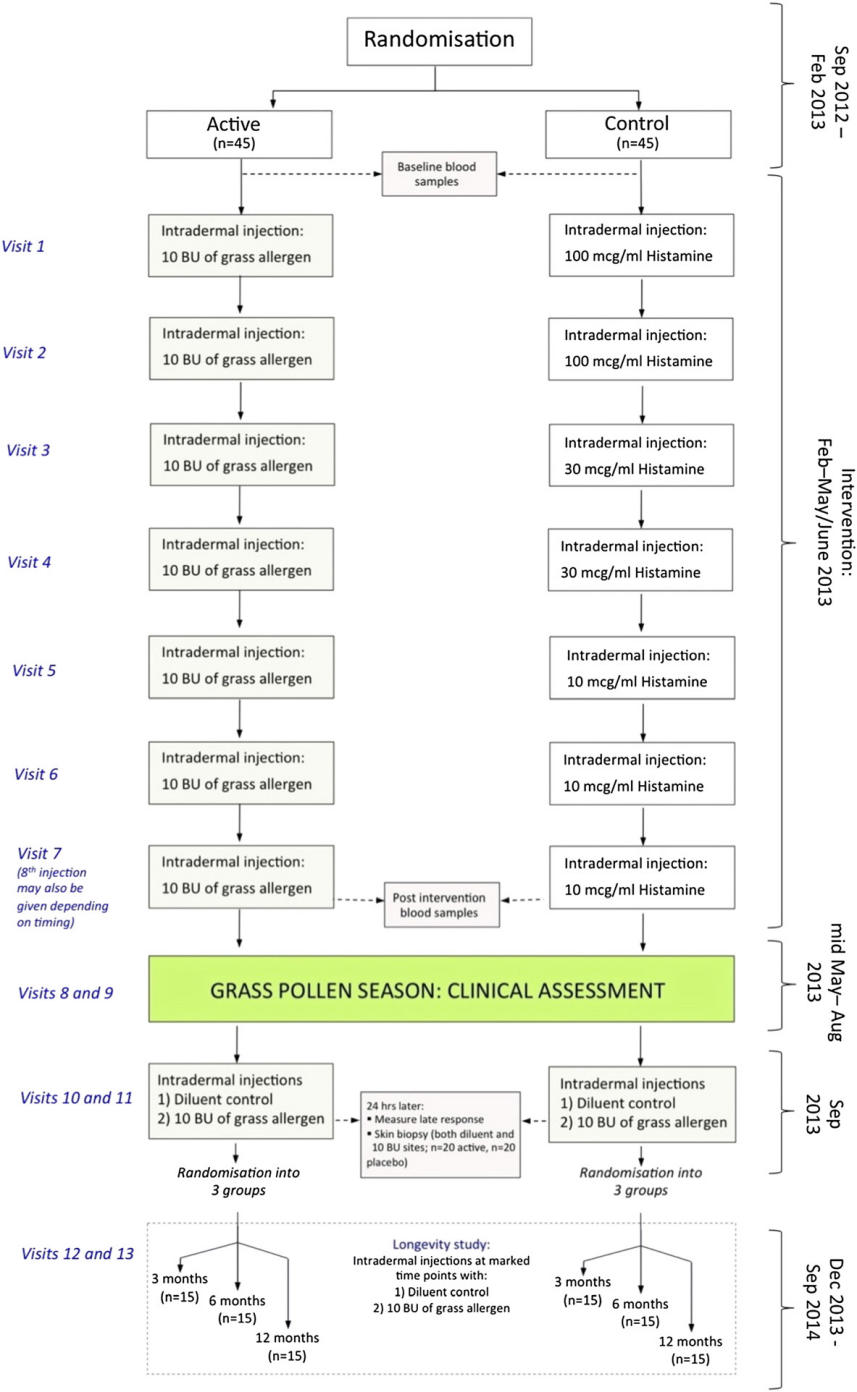
Study design.

**Figure 2:**
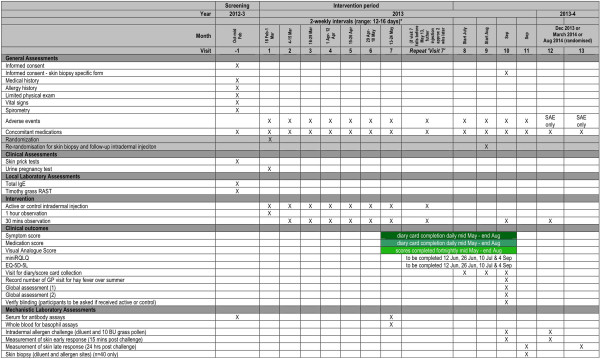
Study schedule.

PollenLITE is a single center (UK) double-blind randomised parallel group controlled trial of the efficacy and safety of 7 to 8 pre-seasonal intradermal injections of *Phleum pratense* grass pollen extract (each containing estimated 7 ng of major allergen Phl p 5) versus histamine control (Figures 1 and 2). The study is based at Guy’s Hospital, King’s College London. A minimum of 90 participants will be recruited and randomised 1:1 to receive active or control injections before the 2013 grass pollen season, when the clinical outcome data will be collected. All participants will also have access to conventional pharmacotherapy for allergic rhinitis. All participants will be consented in accordance with the Declaration of Helsinki. The study has been approved by the London Harrow Research Ethics Committee (reference 12/LO/0941).

### Primary endpoint

Combined symptom and medication score covering the grass pollen season period of 13th May-end August 2013.

### Secondary endpoints

1. Symptom score for each participant, covering the grass pollen season period of 13th May-end August 2013.

2. Medication score for each participant, covering the peak grass pollen season period of 13th May-end August 2013.

3. Quality of life scores, as measured by the mini Rhinoconjunctivitis Quality-of-Life Questionnaire (32) and the EQ-5D-5L questionnaire, during the peak grass pollen season.

4. A Visual Analogue Score for each participant, covering the peak grass pollen season period (mid May-end Aug 2013).

5. A global evaluation by each participant, at the end of the 2013 grass pollen season, of symptoms and a comparison with previous years.

6. Number of primary care (i.e. general practitioner) visits for hay fever during summer 2013.

7. Combined symptom and medication score during the *peak* of the 2013 grass pollen season.

8. Number of medication free days covering the grass pollen season period of 13th May-end August 2013.

9. Number of symptom free days covering the grass pollen season period of 13th May-end August 2013.

10.  Individual symptoms scores (AUC) for each organ: nose, mouth, eyes and lungs.

11.  Total number of days during which prednisolone used between 13th May-end August 2013.

12.  Frequency of adverse events, including the occurrence of systemic allergic reactions.

### Secondary endpoints in mechanistic assays

1) Inflammatory cells (eosinophils, mast cells, basophils, CD4+ T cells and Foxp3+ T cells) in skin biopsies collected 24 hours after receiving an intradermal diluent (negative control) and grass pollen allergen injection in September 2013.

2) Numbers of CRTH2^pos^CD203c^pos^CD3^neg^CD303^neg^ and CRTH2^pos^CD63^pos^CD3^neg^CD303^neg^ activated peripheral blood basophils cells following in vitro activation with grass pollen allergen (Visit 7).

3) Serum concentrations of *Phleum pratense*-specific IgG, IgG1, IgG4 and IgE at Visit 1 and Visit 7. These sera will also be tested for inhibitory activity against allergen-IgE complex binding to B cells.

4) Gene expression profiles of CD4+ T cells derived from skin biopsy explants in September 2013.

5) Cutaneous allergen-induced late response size measured in December 2013 (i.e. approx. 3 months after previous intradermal injection), March 2014 (6 months) or August 2014 (11–12 months).

### Definition of end of study

The duration of the trial is 2 years. The trial will end when the last subject makes the last visit to determine the late response following the final open label follow up intradermal injection at the August 2014 time point.

### Subject selection and withdrawal

#### Inclusion criteria

1) Adults aged 18 to 65 years.

2) A clinical history of grass pollen-induced allergic rhinoconjunctivitis for at least 2 years with peak symptoms in May, June, or July.

3) A clinical history of moderate-severe persistent rhinoconjunctivitis symptoms interfering with usual daily activities or with sleep.

4) A clinical history of rhinoconjunctivitis that remains troublesome despite treatment with either antihistamines or nasal corticosteroids during the grass pollen season.

5) Positive skin prick test response, defined as wheal diameter greater than or equal to 3 mm, to *Phleum pratense*.

6) Positive specific IgE, defined as greater than or equal to IgE class 2, against *Phleum pratense*.

7) For women of childbearing age, a willingness to use an effective form of contraception for the duration of intradermal injections.

8) The ability to give informed consent and comply with study procedures.

#### Exclusion criteria

1) Pre-bronchodilator FEV1 less than 70% of predicted value at screening visit.

2) A history of seasonal grass pollen-induced asthma requiring regular treatment with salbutamol or inhaled corticosteroids. Patients with mild seasonal grass pollen-induced asthma may be included, provided symptoms are satisfactorily controlled with occasional salbutamol only.

3) A clinical history of symptomatic seasonal allergic rhinitis and/or asthma due to tree pollen or weed pollen near or overlapping the grass pollen season, although patients with mild intermittent symptoms requiring only occasional antihistamines may be included.

4) A clinical history of symptomatic allergic rhinitis and/or asthma caused by a perennial allergen to which the participant is regularly exposed, although patients with mild intermittent symptoms requiring only occasional antihistamines may be included.

5) Emergency department visit or hospital admission for asthma in the previous 12 months.

6) History of chronic obstructive pulmonary disease.

7) History of significant recurrent acute sinusitis, defined as 2 episodes per year for the last 2 years, all of which required antibiotic treatment.

8) History of chronic sinusitis, defined as a sinus symptoms lasting greater than 12 weeks outside the grass pollen season, that includes 2 or more major factors or 1 major factor and 2 minor factors. Major factors are defined as facial pain or pressure, nasal obstruction or blockage, nasal discharge or purulence or discolored postnasal discharge, purulence in nasal cavity, or impaired or loss of smell. Minor factors are defined as headache, fever, halitosis, fatigue, dental pain, cough, and ear pain, pressure, or fullness.

9) At randomisation, current symptoms of, or treatment for, upper respiratory tract infection, acute sinusitis, acute otitis media, or other relevant infectious process; serous otitis media is not an exclusion criterion. Participants may be re-evaluated for eligibility after symptoms resolve.

10) Current smokers or a history of greater than or equal to 5 pack years.

11) Previous treatment by immunotherapy with grass pollen allergen within the previous 5 years.

12) History of life-threatening anaphylaxis or angioedema.

13) Ongoing systemic immunosuppressive treatment.

14) History of intolerance of grass pollen immunotherapy, rescue medications or their excipients.

15) For females of childbearing age a positive serum or urine pregnancy test with sensitivity of less than 50 mIU/mL within 72 hours of first administration of study therapy.

16) Lactating females.

17) The use of any investigational drug within 30 days of the screening visit.

18) Ongoing treatment with leukotriene receptor antagonists, beta-blockers, calcium channel blockers, tricyclic antidepressants, monoamine oxidase inhibitors or anti-IgE monoclonal antibody.

19) The presence of any medical condition that the investigator deems incompatible with participation in the trial.

20) Individuals with insufficient understanding of the trial.

### Recruitment

Participants for PollenLITE will be identified via a recruitment campaign including advertisements in press, online and on public transport. Potential participants will be invited to visit the trial website (http://www.pollenlite.co.uk) and answer 7 pre-screening questions before registering. Participants passing pre-screening on the trial website will be contacted for further telephone screening, and if considered potentially suitable will be invited to attend the Clinical Research Facility at Guy’s Hospital for a formal screening visit. A Participant Information Sheet will be provided to each person to read prior to the screening visit. At the screening visit (Visit −1), there will be an opportunity to ask questions of a staff member trained in all trial procedures, as formally delegated by the PI. Written informed consent will be obtained by a physician prior to screening and any other study specific procedures taking place. The original signed consent form will be retained in the Investigator Site File, with a copy filed in the participant’ s hospital records, and a further copy provided to the participant. Individuals will be free to decline further participation without giving reasons. The screening visit will comprise a full medical and allergic history, skin prick testing to a panel of common inhalant allergens, recording of concomitant medications, limited physical examination, measurement of vital signs, spirometry and collection of a venous blood sample for total and *Phleum pretense*-specific IgE levels and for storage for serum based mechanistic assays (baseline sample). For participants who either do not meet the eligibility criteria at the screening visit or decline to proceed further, reasons for non-participation will be recorded where known. The history and test results of every potentially eligible participant will be reviewed by the PI before an appointment is made for Visit 1 (randomisation and first injection). To ensure that a minimum of 90 participants is randomised, up to 100 screened participants will be booked for visit 1, allowing for a 10% drop-out rate between screening and randomisation. In the event that more than 90 eligible participants attend for visit 1, all will be included in the study and randomised up to a maximum of 100. All female participants of childbearing age will be required to undergo a urine pregnancy test with sensitivity of less than 50 mIU/mL within 72 hours of randomisation and first administration of study therapy at Visit 1.

### Trial medication

The active drug will be 10 Biological Units (BU) (33.3 SQ-U) of *Phleum pratense* soluble grass pollen extract (Aquagen SQ Timothy, ALK Abello, Reading UK) contained in a 20 microliter volume (i.e. 500 BU/ml (1666.7 SQ-U/ml)). Individual vials for each participant and each visit will be pre-prepared and pre-labeled by Guy’s Hospital Pharmacy under GMP conditions. In brief, Aquagen SQ Timothy Grass Pollen extract will be reconstituted in manufacturer-supplied diluent to the maximum recommended concentration (30'000 BU/ml (100'000 SQ-U/ml) i.e. 60-times final working strength; shelf life 6 months at 2-8°C after reconstitution) and 0.15 ml aliquoted into glass study vials. At each visit for intradermal injection the investigator will add 8.85 ml of clinical grade 0.9% normal saline at ambient temperature to the vial corresponding to that participant’s visit to achieve a 60-fold dilution. Twenty microliters will then be aspirated from this vial and administered directly. The allergen requires dilution on the day of administration as the recommended shelf life of Aquagen SQ Timothy Grass Pollen extract at 500 BU/ml (1666.7 SQ-U/ml) is only 14 days. Control drug will be histamine only, administered at a concentration of 100 mcg/ml. To help preserve blinding, histamine concentrations will be reduced to 30 mcg/ml for the 3^rd^ and 4^th^ injections, and 10 mcg/ml for 5, 6 and 7^th^ injections. To match the grass pollen extract dilution and preserve blinding, histamine will also be aliquoted into study vials at 60-times final working strength in 0.15 ml volumes, for further dilution with 8.85 ml of clinical grade 0.9% normal saline immediately prior to injection. Active and control study medications will appear identical.

Following manufacture, vials will be packed into individual dispensing packs that will be dispensed by pharmacy against a single study prescription for each study participant, covering all visits. At randomisation, an email will be sent from the randomisation system to the dispensing pharmacy. On receipt of a study prescription, the pharmacist will refer to this email to select an appropriate pack of active or control medication. The pack will then be fully blinded and dispensed. The blinded dispensed packs will thereafter be stored in the Clinical Research Facility in temperature monitored fridges in a secure environment.

### Dosing regimen

A series of 7 intradermal active or control injections will be administered 2-weekly into the forearm between before the 2013 grass pollen season. The first injection will be administered between 18th February and 1st March 2013. We will aim to administer the 7th injection between 13th May and 24th May 2013. The injection site will again be alternated between left and right arms at each visit. Intradermal injections will be administered in a 20 microliter volume using a 29 gauge insulin syringe (Becton Dickinson Micro-FineTM). As a precaution against systemic allergic reactions all subjects will be observed for one hour after the first injection, and for 30 minutes after subsequent injections, in a clinical area with full resuscitation facilities.

Occasionally an injection may be administered too deeply (into subcutaneous tissue) resulting in failure to generate an immediate injection ‘bleb’ and subsequent characteristic wheal. For this reason, the injection site will be inspected in all subjects 15 minutes after each injection to confirm successful intradermal injection. In the absence of the typical wheal, the injection will be repeated at 1 cm from the original site.

In the event of acute illness (e.g. upper respiratory tract infection) or other unforeseeable circumstance, administration of active/control intervention may be postponed up to a maximum of 3 weeks since the previous injection, at the discretion of the investigator. The study team aim to perform over 90% of injections at 12–16 day intervals, although in exceptional circumstances the minimal interval permitted between injections may be reduced to 7 days. Onset of symptoms associated with the London grass pollen season generally falls between late May - early June. Therefore, if schedule adjustments result in a participant completing their 7 injections before 13th May 2013, the 7th injection will be repeated 12–16 days later in late May.

Following these injections, clinical outcomes will be measured during the summer of 2013. In September 2013 and either 3, 6 or 12 month later, all participants will also receive intradermal injections with diluent (negative control) or 10 BU (33.3 SQ-U) of grass pollen allergen. Although the trial will not be unblinded at this stage, these intradermal injections will be open label.

Most participants will not be taking antihistamines at the time of intradermal injections since these will be performed before the grass pollen season. Nevertheless all participants will be asked to avoid taking antihistamines for 5 days before receiving an intradermal injection so that the presence of a wheal can be confirmed. Following an intradermal injection participants may take an antihistamine to reduce the local itching and swelling. If they wish to do so before leaving the clinical research facility an antihistamine tablet will be provided. The exception will be visits 10 and 11, when cutaneous response sizes will be measured and therefore participants will be asked to refrain from taking an antihistamine. Any oral steroids must be stopped for a minimum of 2 weeks before the intradermal injection in September 2013 since this may interfere with late response measurements and skin biopsy studies. Participants will be asked to refrain from alcohol consumption on the same day before and for 2 hours after an intradermal injection.

### Study medication accountability

Study drug accountability will be assessed and documented by Guy’s Hospital Pharmacy. Study vials that have been reconstituted in saline for injection and used will be stored separately at room temperature after use for return to pharmacy for drug accountability to be assessed.

### Concomitant medications

Rescue medications will be provided to participants before and throughout the pollen season. These will include: desloratadine (5 mg, up to 1 tablet daily), (olopatadine eye drops, 1.0 mg/mL, up to 1 drop per eye twice daily), fluticasone propionate nasal spray 50 mcg per spray, up to 2 sprays per nostril once daily), and prednisone (for use at 30 mg per day for up to 5 days). Participants will be asked to use only these medications to treat their hay fever symptoms on an as required basis. However, participants who are not getting hay fever symptoms will be encouraged to try not to use these medications. Participants will be asked to use only these medications. A short course of prednisolone will be available if symptoms are particularly severe. Participants will be instructed to contact a trial physician prior to taking any prednisolone. The doctor will then provide instructions on dose and duration of treatment. Concurrent treatment with beta-blockers, calcium channel blockers, tricyclic antidepressants, monoamine oxidase inhibitors or anti-IgE monoclonal antibody will not be permitted.

### Randomisation

The King’s Clinical Trial Unit (KCTU) at King’s College London will host a 24 hour web based randomisation system. Participants will be randomised 1:1 to active and comparator medications by the method of block randomisation with randomly varying block sizes, stratified by the size of skin test response to grass pollen at screening visit (the cut-off skin prick test size will be the median value of all subjects to be randomised) and presence/absence of rhinitis symptoms outside the grass pollen season. Study medication will be blinded. To minimise bias through accidental unblinding as a result of common injection site reactions in the active trial arm, the control intervention will consist of a reducing dose of histamine, which will produce similar clinical effects as the active medication. All physicians, researchers, research nurses, outcome assessors and patients will remain blinded to treatment allocation until the primary analysis is completed. The trial statistician will be sub-group unblind only. The KCTU randomisation service provider and the manufacturing pharmacy only will have access to the blinding information for the study.

In August 2013, KCTU will randomly select participants to be approached in rotation to undergo skin biopsies. The first 40 participants who agree will then undergo biopsy after giving additional procedure-specific informed consent. Also in August 2013, KCTU will randomise all participants for a second time to one of three groups (3 months, 6 months or 12 months). These 3 groups will then undergo repeat intradermal allergen injections at 3, 6 or 12 months, respectively, to assess if low dose intradermal allergen immunotherapy is associated with prolonged suppression skin responses.

### Withdrawal of participants

The study therapy (active or control) will be discontinued for any of the following reasons:

1. Inability or failure to attend for intervention within 3 weeks of previous administration.

2. Inability or failure to receive 7 or 8 injections within the dates specified.

3. Two Grade 2 systemic reactions, or a single systemic reaction of Grade 3 or above after administration of study therapy. Systemic reactions will be graded according to the World Allergy Organization (WAO) criteria:

•Grade 1: symptoms of 1 organ system (cutaneous, upper respiratory tract, conjunctival, gastrointestinal, other);

•Grade 2: symptoms of more than 1 organ system present or asthma symptoms/signs (cough, wheezing, shortness of breath but < 40% drop in peak expiratory flow [PEF] or FEV1).

•Grade 3: asthma symptoms/signs (with ≥ 40% drop in PEF or FEV1), upper respiratory tract (laryngeal, uvula, tongue) oedema with or without stridor.

•Grade 4: respiratory failure or hypotension with or without loss of consciousness.

4. An adverse event that, in the judgment of the principal investigator or the medical monitor, presents an unacceptable consequence or risk to the participant.

5. An illness or infection that is not associated with the condition under study and that requires treatment not consistent with protocol requirements; or, if a participant develops an intercurrent illness that in the judgment of the principal investigator in any way justifies discontinuation.

6. An inability or unwillingness to comply with the study protocol, and the protocol deviations are sufficient to jeopardise the participant’s well-being or the integrity of the study.

7. Pregnancy occurs during study participation.

Participants have the right to withdraw from the study at any time for any reason. The investigator also has the right to withdraw patients from the study drug in the event of inter-current illness, AEs, SAE’s, SUSAR’s, protocol violations, cure, administrative reasons or other reasons. As an excessive rate of withdrawals could render the study uninterpretable unnecessary withdrawal of patients will be avoided where possible. Should a participant decide to withdraw from the study, all efforts will be made to report the reason for withdrawal as thoroughly as possible. Should a participant withdraw from study drug only, efforts will be made to continue to obtain follow-up data, with the permission of the patient. A 10% allowance has been included in the sample size to account for withdrawal and participants will only be replaced if this occurs prior to randomisation. The statistical analysis also incorporates a plan for dealing with missing data from symptom and medication diary cards.

### Assessment of efficacy

#### Primary efficacy parameters

The primary outcome measure will be daily symptoms and medication use during the 2013 grass pollen season. The scoring systems have been adapted from previous trials of grass pollen immunotherapy [29]. Participants will record symptom scores each day (reflecting the preceding 24 hours) in diary cards from mid-May through to the end of August. The symptom score will be based on individual symptoms in the nose (sneezing, blockage, and running), eyes (itching, redness, tears, and swelling), mouth and throat (itching and dryness), and chest (breathlessness, cough, wheezing, and tightness), recorded on a scale of 0 to 3 (with a score of 0 indicating no symptoms and 1, 2, and 3 indicating mild, moderate, and severe symptoms, respectively). The maximum daily symptoms score will therefore be 39. All possible rescue medications will be provided to each participant approximately 2 weeks before and throughout the pollen season. Participants will be asked to use medications to treat symptoms on an as required basis. Daily medication use will also be recorded in diary cards by participants and a medication score calculated based on use according to need of the following medications: desloratadine, 5 mg, up to 1 tablet daily (6 points per day); olopatadine eye drops, 1.0 mg/mL, up to 1 drop per eye twice daily (1.5 points per drop, up to 6 points per day); fluticasone nasal spray, 50 mcg per spray, up to 2 sprays per nostril once daily (2 point per spray, up to 8 points per day); and prednisone, 5 mg per tablet, up to 6 tablets per day (2 points per tablet, up to 12 points per day). All these medications will be provided to participants free of any charge. The maximum daily medication score will therefore be 32. For each participant, both the symptom score and the medication score will be individually expressed as area under curve (AUC) values for the period corresponding to the grass pollen season (mid May - August 2013). Since maximum scores for symptoms and medications are different in magnitude these parameters will be normalised as recommended World Allergy Organization guidelines [30]. Normalised AUC scores will then be added together to generate a combined symptom and medication score in accordance with World Allergy Organization guidance on immunotherapy trials [30]. Efficacy will then be assessed by comparison of this combined score in active and control groups.

#### Secondary efficacy parameters

The following will be compared in active and control groups:

1) Symptom scores (AUC) calculated as above.

2) Medication scores (AUC), calculated as above.

3) Rhinoconjunctivitis Quality of Life: mini Rhinitis Quality of Life Scores (RQLQ) scores (overall score and domain scores) will be recorded three times during the pollen season (June 12, June 26 and July 10) and once after the season on 4 September 2013. These values will be compared in active and control groups. The mini RQLQ covers five dimensions of health including sleep, non-nose/eye symptoms, practical problems, nasal symptoms, eye symptoms.

4) Health related quality of life: This will be evaluated using the EQ-5D-5L questionnaire three times during the pollen season (June 12, June 26 and July 10) and once after the season on 4 September 2013.

5) Visual Analogue Scores (see Additional file 1). These will be recorded every 2 weeks during the pollen season and AUC values calculated.

6) Global evaluation scores (see Additional file 2).

7) The number of primary care (i.e. general practitioner) visits for hay fever during summer 2013.

8) Combined symptom and medication scores during the peak* of the 2013 grass pollen season.

9) Number of medication free days covering the grass pollen season period of 13th May-end August 2013 will be compared in active and control groups.

10) Number of symptom free days covering the grass pollen season period of 13th May-end August 2013 will be compared in active and control groups.

11) Individual symptoms scores (AUC) for each organ: nose, mouth, eyes and lungs.

12) Total number of days during which prednisolone used between 13th May-end August 2013.

*The peak of grass pollen season will be defined as starting on the first 3 consecutive days between 13 May and 31 August 2013 when grass pollen counts in central London are ≥ 30 grains/cm^3^, using counts supplied by the UK Met Office. The end of the peak season will be defined as the first of 3 consecutive days when grass pollen counts are < 30 grains/cm^3^. In the event of 2 or more peaks during the 2013 season, these individual peak periods will be analysed separately.

The persistence of late response suppression will be evaluated by randomising active and control subjects in Aug 2013 into 3 groups. Each group will receive a follow-up intradermal injection of 10 BU (33.3 SQ-U) of grass pollen allergen, as well as a diluent control injection, at either 3, 6 or 12 months. Reversal of late response suppression will be assessed at each time point by comparing mean late response size in the active and control groups.

### Procedures for assessing efficacy parameters

#### Primary efficacy parameters

All participants will be supplied with diary cards. They will be asked to score symptoms and medication use for each day. Participants will be asked to provide a copy of the first diary card (corresponding to 13–30 May 2013) during the first week of June by post, fax or email. Thereafter original cards will be collected approximately monthly for transcription to the eCRF. Participants will be contacted with regular reminders to complete the cards. As a further precaution against lost data, every participant will be individually contacted by telephone, email or in person to provide 24 hour dairy card data for 13^th^ May, 1st July and 31^st^ August 2013 to be used to assist in the statistical imputation of missing data in the event of incomplete data.

#### Secondary efficacy parameters

The Visual Analogue Scores will require completion every 2 weeks over the summer. Participants will be reminded to complete these as above and these will be collected at the same visits. Mini RQLQ and EQ-5D-5 L forms will be completed three times during the pollen season (June 12, June 26 and July 10) and once after the season on 4 September 2013. These forms will be collected at visits 8, 9 and 10.

### Safety

#### Specification, timing and recording of safety parameters

All adverse events and side effects will be recorded in the electronic case report form (eCRF) throughout the study regardless of their severity or relation to study participation.

Systemic allergic reactions to grass pollen intradermal injections are a theoretical possibility. As a precaution, all participants in this trial will be observed after the first intradermal injection for one hour, and if there is no systemic reaction, for 30 minutes after subsequent injections (since the dose of allergen given does not increase at later visits). All injections will be given in a clinical area with resuscitation facilities in the presence of a physician. If a participant has a Grade 2 reaction (see above) they may continue in the study but the observation period will be increased to 1 hour after each subsequent injection. In the event of a further Grade 2 reaction, that participant will not be permitted to continue in the study. In the event of a participant experiencing a Grade 1 reaction, the clinical observation period for that individual will also be maintained at 1 hour after subsequent injections. In the event of a single Grade 3 or 4 reaction, that participant will not be permitted to continue in the study.

#### Procedures for recording and reporting adverse events

These details are outlined in Additional file 3.

#### Adverse events that do not require reporting

These will include the following:

1) Symptoms due to aeroallergen exposure i.e. nasal blockage, rhinorrhea, itching or sneezing; Itching, watering redness or swelling of eyes; itching or dryness of mouth/throat; breathless, cough, wheeze and chest tightness.

2) Transient discomfort from intradermal injections.

3) Appearance of an itchy oedematous wheal with surrounding erythema (early response) after intradermal injection.

4) Appearance of swelling (oedema) within hours of intradermal injection, that may persist for several days (late response). These responses may be associated with very mild local tenderness.

5) Temporary discomfort,, bleeding, bruising, swelling at the needle site following venesection, and in rare instances, infection. Some people may experience lightheadedness, nausea, or fainting.

6) Mild localised itching arising from skin prick testing during screening.

#### Treatment stopping rules

The trial will be stopped in the event of five grade 3 reactions or a single grade 4 reaction. In addition the trial may be prematurely discontinued by the Sponsor, Chief Investigator or Regulatory Authority on the basis of new safety information or for other reasons given by the Data Monitoring & Ethics Committee/Trial Steering Committee regulatory authority or ethics committee. The trial may also be prematurely discontinued due to lack of recruitment or upon advice from a Trial Steering Committee who will advise on whether to continue or discontinue the study and make a recommendation to the sponsor. If the study is prematurely discontinued, participants will be informed and no further participant data will be collected.

#### Code breaking

24 hr Emergency Code Break and Medical Information will be provided by Guy’s & St Thomas’ NHS Foundation Trust Emergency Scientific Medical Services (eSMS). Each randomised subject will be provided with a card detailing code break telephone numbers and emergency contact details. Subjects will be requested to carry this card with them at all times whilst participating in the trial.

### Study data

#### Data handling

The Chief Investigator will act as custodian for the trial data. The following guidelines will be strictly adhered to:

•Patient data will be anonymised.

•All anonymised data will be stored on a password protected computer.

•All trial data will be stored in line with the Medicines for Human Use (Clinical Trials) Amended Regulations 2006 and the Data Protection Act and archived in line with the Medicines for Human Use (Clinical Trials) Amended Regulations 2006 as defined in the King’s Health Partner’s Clinical Trials Office Archiving SOP.

#### Data management

Data will be managed using the InferMed MACRO database system. An electronic Case Report Form (eCRF) will be created using the InferMed Macro system. This system is regulatory compliant (GCP, 21CRF11, EC Clinical Trial Directive). The eCRF will be created in collaboration with the trial statisticians and the CI and maintained by the King’s Clinical Trials Unit. It will be hosted on a dedicated secure server within KCL. Source data will be entered by authorised staff onto the eCRF with a full audit trail.

#### Database passwords

Database access will be strictly restricted through passwords to the authorised research team. The CI or delegate will request usernames and passwords from the KCTU. It is a legal requirement that passwords to the eCRF are not shared, and that only those authorised to access the system are allowed to do so. If new staff members join the study, a personalised username and password will be requested via the CI or delegate.

#### Data handling & confidentiality/format of records

Data will be handled, computerised and stored in accordance with the Data Protection Act, 1998. Participants will be identified on the study database using a unique code and initials. The investigator will maintain accurate patient records/results detailing observations on each patient enrolled.

#### Identifiable data

All participant contact information data will be stored on spreadsheets within the recruiting NHS site, which will have restricted access from password protected computers. Accrual data uploaded to the UKCRN portfolio database will be anonymised and collated by the CI or delegate to the CLRN. No identifiable data will be entered on the eCRF or transferred to the KCTU.

#### Main database

SAE data will be collected on paper SAE report forms and faxed to the KHPCTO. Summary details of SAEs will be transcribed to adverse event section of the eCRF. For all other data collected, source data worksheets will be prepared for each patient and data will be entered onto the eCRF database. Source data worksheets will be reconciled at the end of the trial with the patients NHS medical notes in the recruiting center. During the trial, critical clinical information will be written in the medical notes to ensure informed medical decisions can be made in the absence of the study team. Trial related clinical letters will be copied to the medical notes during the trial. The Chief Investigator will provide an electronic signature for each patient Case Record Form once all queries are resolved and immediately prior to database lock.

At the end of the study, essential documentation will be archived in accordance with sponsor and local requirements. The retention of study data will be the responsibility of the Chief Investigator. Any data exports (other than baseline data) will require authorisation by the trial statistician.

#### Statistical analysis

The Statistical Analysis Plan will be finalised by the trial statistician and approved by the TSC and the DMEC prior to database lock. The study will be unblinded after the final intradermal injection in September 2014. No interim analysis is planned although pre-defined stopping criteria will be discussed by the TSC and the Independent DMEC and agreed if appropriate. Descriptive statistics will be produced for DMEC reports and in the primary analysis. For each of the variables analysed, univariate descriptive statistics will be summarised by randomised group to provide an overview of the data. Summary measures for the baseline characteristics of each group will be presented as mean and standard deviation for continuous (approximate) normally distributed variables, medians and interquartile ranges for non-normally distributed variables, and frequencies and percentages for categorical variables. The Area under the Curves (AUC) of the combined symptom and medication scores for the period corresponding to the grass pollen season (mid May-Aug) will be plotted against time as a summary measure of the primary outcome. This will provide each patient’s longitudinal outcome as a single quantity, which will be calculated for Symptom and Medication scores. The planned primary efficacy analysis i.e. the difference between the two arms in AUC of the combined symptom and medication scores, will be analysed on randomised patients using (stratified) Mann–Whitney *U* test (Van Elteren test statistic), adjusted for the baseline stratification factors (size of the skin test to grass pollen and presence or absence of rhinitis symptoms outside the grass pollen season). Sample size estimation assumed 10% of patients would not provide evaluable end of study information. If this rate is observed, multiple imputation methods will be used in order to provide an overall treatment effect estimate with a standard error that is properly inflated to incorporate uncertainty associated with imputing values. Since this may introduce a bias if the main reason for drop-out was deterioration, a sensitivity analysis will be performed to explore departures from the ‘missing at random’ assumption. Similar analyses will be conducted for secondary (symptom scores, medication scores and individual symptoms) and mechanistic outcomes. Regression models will be also used to evaluate the change in RQLQ scores to isolate the effect of the intervention on each arm after adjusting for stratification factors. In analyzing the recovery of the cutaneous late response at each 3, 6 and 12 month time point, the size of late response in the group that originally received active therapy will be compared with the group that originally received the control intervention. As a further sensitivity analysis, all key outcomes will be re-analysed adjusting for any observed differences at baseline that are judged to be of clinical importance. Differences between the groups will be estimated with 95% confidence intervals. The principal software package will be SAS, with verification of results from syntax for selected analyses analysed in STATA.

#### Sample size calculation

Power calculations for the primary outcome (combined symptom and medication score) were performed based on raw data from a previous clinical trial of subcutaneous grass pollen immunotherapy [29]. The power calculation has been conservatively based on the detection of a clinical effect size 80% of that reported in that trial. Using this method and a two-sided non-parametric test based on a Monte Carlo approach, group sample sizes of 35 and 35 achieve 90% power to detect such difference in AUC of the combined symptom and medication scores at a significance level of 0.05. To make allowance for the unknown distribution of the primary outcome and based on the lower bound for the asymptotic relative efficiency (ARE) of the Mann–Whitney *U* test, we have increased the sample size by a further 15% to 40 in each arm. Further accounting for a post-randomisation dropout rate of up to 10% consistent with previous trials of grass pollen immunotherapy, a total sample size of 90 (45 each arm) is required. Recruitment will take place several months before visit 1. At visit 1 randomisation will be performed and the first injection administered. To ensure that a minimum of 90 participants is randomised, up to 100 screened participants will be booked for visit 1, allowing for a 10% drop-out rate between screening and randomisation.

## Discussion

This protocol will allow us to test our hypothesis that low dose intradermal immunotherapy with grass pollen allergen is an effective treatment in seasonal allergic rhinitis caused by grass pollen. The first participant was randomised in February 2013 and the last participant is expected to complete the trial protocol in August 2014.

## Abbreviations

AE: Adverse event; AR: Adverse reaction; CI: Chief investigator; DMEC: Data monitoring committee; DSUR: Development safety update reports; eCRF: Electronic case record form; eSMS: Emergency scientific & medical services; EudraCT: European union drug regulating authorities clinical trials; FEV1: Forced expiatory volume; GCP: Good clinical practice; IgE: Immunoglobulin E; IMP: Investigational medicinal product; ISRCTN: International standardized randomised controlled trial number; KCL: King’s college london; KCTU: King’s clinical trials unit, King’s college london (UKCRC registered KCTU); KHP-CTO: Kings health partners clinical trials office (function of the sponsor); MHRA: Medicines & healthcare products regulatory agency; NIHR: National institute for health research; NRES: National research ethics service; PEF: Peak expiratory flow; PollenLITE: Pollen Low dose Intradermal Therapy Evaluation; REC: Research ethics committee; SAE: Serious adverse event; SAR: Serious adverse reaction; SOP: Standard operating procedure; SPC/SmPC: Summary of product characteristics; SUSAR: Suspected unexpected serious adverse reaction; TSC: Trial steering committee; UKCRN: UK clinical research network.

## Competing interests

SJT and SRD have both received research funding from ALK Abello.

## Authors’ contribution

SJT is chief investigator of the PollenLITE trial and conceived of the study, participated in its design, co-ordination and prepared the first draft of this manuscript with the assistance of AS. AS, AG, KT and RM participated in the set up of the trial, recruitment and administration of intradermal injections. AD participated in the design of the trial and is the trial statistician. JK and CM participated in the design and set up of the trial. SRD participated in the study conception and design. MS and SY participated in design and co-ordination of immune assays. All authors read and approved the final manuscript.

## Supplementary Material

Additional file 1 **Visual Analogue Score.** To be completed every 2 weeks over Summer 2013. Click here for file

Additional file 2 Global evaluation scores: to be completed September 2013. Click here for file

Additional file 3 Procedures for Recording and Reporting Adverse Events. Click here for file

## References

[B1] Bauchau V Durham SR Prevalence and rate of diagnosis of allergic rhinitis in Europe Eur Respir J 2004 24 758 764 10.1183/09031936.04.00013904 15516669

[B2] Bauchau V Durham SR Epidemiological characterization of the intermittent and persistent types of allergic rhinitis Allergy 2005 60 350 353 10.1111/j.1398-9995.2005.00751.x 15679721

[B3] Walker SM Durham SR Till SJ Roberts G Corrigan CJ Leech SC Krishna MT Rajakulasingham RK Williams A Chantrell J Immunotherapy for allergic rhinitis Clin Exp Allergy 2011 41 1177 1200 10.1111/j.1365-2222.2011.03794.x 21848757

[B4] Noon L Prophylactic inoculation against hay fever Lancet 1911 1 1572 1573

[B5] Calderon MA Alves B Jacobson M Hurwitz B Sheikh A Durham S Allergen injection immunotherapy for seasonal allergic rhinitis Cochrane Db Syst Rev 2007 1 CD001936 10.1002/14651858.CD001936.pub2 PMC7017974 17253469

[B6] Radulovic S Calderon MA Wilson D Durham S Sublingual immunotherapy for allergic rhinitis Cochrane Db Syst Rev 2010 12 CD002893 10.1002/14651858.CD002893.pub2 PMC7001038 21154351

[B7] Rotiroti G Shamji M Durham SR Till SJ Repeated low-dose intradermal allergen injection suppresses allergen-induced cutaneous late responses J Allergy Clin Immunol 2012 130 918 924 e911 10.1016/j.jaci.2012.06.052 22971521

[B8] Francis JN James LK Paraskevopoulos G Wong C Calderon MA Durham SR Till SJ Grass pollen immunotherapy: IL-10 induction and suppression of late responses precedes IgG4 inhibitory antibody activity J Allergy Clin Immunol 2008 121 1120 1125 e1122 10.1016/j.jaci.2008.01.072 18374405

[B9] Lima MT Wilson DR Pitkin L Roberts A Nouri-Aria KT Jacobson M Walker SM Durham SR Grass pollen immunotherapy (SLIT) for seasonal rhinoconjunctivitis: a randomised controlled trial Clin Exp Allergy 2001 31 1158 1158 10.1046/j.0954-7894.2002.01327.x 11972594

[B10] Phillips EW Relief of hay-fever by intradermal injections of pollen extract J Amer Med Assoc 1926 86 182 184 10.1001/jama.1926.02670290022008

[B11] Phillips EW Intradermal pollen therapy during the attack J Allergy 1933 5 29 36 10.1016/S0021-8707(33)90167-7

[B12] Francis JN Till SJ Durham SR Induction of IL-10 + CD4 + CD25+ T cells by grass pollen immunotherapy J Allergy Clin Immunol 2003 111 1255 1261 10.1067/mai.2003.1570 12789226

[B13] Jutel M Akdis M Budak F Aebischer-Casaulta C Wrzyszcz M Blaser K Akdis CA IL-10 and TGF-beta cooperate in the regulatory T cell response to mucosal allergens in normal immunity and specific immunotherapy Eur J Immunol 2003 33 1205 1214 10.1002/eji.200322919 12731045

[B14] Radulovic S Jacobson MR Durham SR Nouri-Aria KT Grass pollen immunotherapy induces Foxp3-expressing CD4+ CD25+ cells in the nasal mucosa J Allergy Clin Immunol 2008 121 1467 1472 1472 e1461 10.1016/j.jaci.2008.03.013 18423565

[B15] Kersey TW Van Eyk J Lannin DR Chua AN Tafra L Comparison of intradermal and subcutaneous injections in lymphatic mapping J Surg Res 2001 96 255 259 10.1006/jsre.2000.6075 11266281

[B16] Senti G von Moos S Kundig TM Epicutaneous allergen administration: is this the future of allergen-specific immunotherapy? Allergy 2011 66 798 809 10.1111/j.1398-9995.2011.02560.x 21518374

[B17] Romani N Flacher V Tripp CH Sparber F Ebner S Stoitzner P Targeting Skin Dendritic Cells to Improve Intradermal Vaccination Curr Top Microbiol Immunol 2011 351 113 138 10.1007/82_2010_118 21253784 PMC4285659

[B18] Varney VA Hamid QA Gaga M Ying S Jacobson M Frew AJ Kay AB Durham SR Influence of Grass-Pollen Immunotherapy on Cellular Infiltration and Cytokine Messenger-Rna Expression during Allergen-Induced Late-Phase Cutaneous Responses J Clin Invest 1993 92 644 651 10.1172/JCI116633 8349803 PMC294897

[B19] Durham SR Ying S Varney VA Jacobson MR Sudderick RM Mackay IS Kay AB Hamid QA Grass pollen immunotherapy inhibits allergen-induced infiltration of CD4+ T lymphocytes and eosinophils in the nasal mucosa and increases the number of cells expressing messenger RNA for interferon-gamma J Allergy Clin Immunol 1996 97 1356 1365 10.1016/S0091-6749(96)70205-1 8648033

[B20] Hamid QA Schotman E Jacobson MR Walker SM Durham SR Increases in IL-12 messenger RNA + cells accompany inhibition of allergen-induced late skin responses after successful grass pollen immunotherapy J Allergy Clin Immunol 1997 99 254 260 10.1016/S0091-6749(97)70106-4 9042055

[B21] Durham SR Varney VA Gaga M Jacobson MR Varga EM Frew AJ Kay AB Grass pollen immunotherapy decreases the number of mast cells in the skin Clin Exp Allergy 1999 29 1490 1496 10.1046/j.1365-2222.1999.00678.x 10520076

[B22] Nasser SMS Ying S Meng Q Kay AB Ewan PW Interleukin-10 levels increase in cutaneous biopsies of patients undergoing wasp venom immunotherapy Eur J Immunol 2001 31 3704 3713 10.1002/1521-4141(200112)31:12<3704::AID-IMMU3704>3.0.CO;2-3 11745391

[B23] Wilson DR Nouri-Aria KT Walker SM Pajno GB O’Brien F Jacobson MR Mackay IS Durham SR Grass pollen immunotherapy: symptomatic improvement correlates with reductions in eosinophils and IL-5 mRNA expression in the nasal mucosa during the pollen season J Allergy Clin Immunol 2001 107 971 976 10.1067/mai.2001.115483 11398073

[B24] Nouri-Aria KT Wachholz PA Francis JN Jacobson MR Walker SM Wilcock LK Staple SQ Aalberse RC Till SJ Durham SR Grass pollen immunotherapy induces mucosal and peripheral IL-10 responses and blocking IgG activity J Immunol 2004 172 3252 3259 14978133 10.4049/jimmunol.172.5.3252

[B25] Nouri-Aria KT Pilette C Jacobson MR Watanabe H Durham SR IL-9 and c-Kit + mast cells in allergic rhinitis during seasonal allergen exposure: effect of immunotherapy J Allergy Clin Immunol 2005 116 73 79 10.1016/j.jaci.2005.03.011 15990777

[B26] Francis JN Shamji MH Wilcock LK Wachholz PA Dearman RJ Kimber I Wurtzen PA Larche M Durham SR The IgE-facilitated allergen binding (FAB) assay: Validation of a novel flow-cytometric based method for the detection of inhibitory antibody responses J Immunol Methods 2006 317 71 79 10.1016/j.jim.2006.09.004 17070537 PMC1934503

[B27] Durham SR Walker SM Varga EM Jacobson MR O’Brien F Noble W Till SJ Hamid QA Nouri-Aria KT Long-term clinical efficacy of grass-pollen immunotherapy N Engl J Med 1999 341 468 475 10.1056/NEJM199908123410702 10441602

[B28] Durham SR Emminger W Kapp A Colombo G de Monchy JG Rak S Scadding GK Andersen JS Riis B Dahl R Long-term clinical efficacy in grass pollen-induced rhinoconjunctivitis after treatment with SQ-standardized grass allergy immunotherapy tablet J Allergy Clin Immunol 2010 125 131 138 e131-137 10.1016/j.jaci.2009.10.035 20109743

[B29] Varney VA Gaga M Frew AJ Aber VR Kay AB Durham SR Usefulness of immunotherapy in patients with severe summer hay fever uncontrolled by antiallergic drugs Brit Med J 1991 302 265 269 10.1136/bmj.302.6771.265 1998791 PMC1668945

[B30] Canonica GW Baena-Cagnani CE Bousquet J Bousquet PJ Lockey RF Malling HJ Passalacqua G Potter P Valovirta E Recommendations for standardization of clinical trials with Allergen Specific Immunotherapy for respiratory allergy. A statement of a World Allergy Organization (WAO) taskforce Allergy 2007 62 317 324 10.1111/j.1398-9995.2006.01312.x 17298350

