# Supplementary material for: Protocol for a double-blind randomised controlled trial of low dose intradermal grass pollen immunotherapy versus a histamine control on symptoms and medication use in adults with seasonal allergic rhinitis (PollenLITE)
Source: Clin Transl Allergy. 2013 Aug 21;3:27. doi: 10.1186/2045-7022-3-27 (PMC3765857; doi:10.1186/2045-7022-3-27)
Supplement: Additional file 1 — Visual Analogue Score. To be completed every 2 weeks over Summer 2013. [file 2045-7022-3-27-S1.pdf]

ADDITIONAL INFORMATION

Visual Analogue Score

To be completed every 2 weeks over Summer 2013

Please place a vertical mark along the line where you feel the severity of your symptoms lie. So, if you were to place a mark on the far left of the line, it would mean that you are completely symptom free. However, if you marked the far right of the line, your symptoms are as bad as they possibly could be.

Nasal Symptoms:

Nasal Blockage/Congestion

____

Runny nose

____

Itchy nose

____

Sneezing

____

Eye Symptoms:

Itchy eyes

____

Watery eyes

____
