# Supplementary material for: Protocol for a double-blind randomised controlled trial of low dose intradermal grass pollen immunotherapy versus a histamine control on symptoms and medication use in adults with seasonal allergic rhinitis (PollenLITE)
Source: Clin Transl Allergy. 2013 Aug 21;3:27. doi: 10.1186/2045-7022-3-27 (PMC3765857; doi:10.1186/2045-7022-3-27)
Supplement: Additional file 2 — Global evaluation scores: to be completed September 2013. [file 2045-7022-3-27-S2.pdf]

ADDITIONAL INFORMATION

Global evaluation scores: to be completed September 2013

Global Evaluation No. 1

Sept 2013 visit

The subject should be asked: "How do you assess the severity of your rhinoconjunctivitis symptoms when they were the most severe during this grass pollen season (Tick each single symptom)?

**Rhinoconjunctivitis/ Hayfever symptom**		**Symptoms**			
		0 (None	1 (Mild)	2 (Moderate)	3 (Severe)
Nasal Symptoms					
1.	Runny nose				
2.	Blocked nose				
3.	Sneezing				
4.	Itchy nose				
Eye symptoms					
1.	Itchy eyes				
2.	Watery eyes				

Global Evaluation No. 2

Sept 2013 visit

The subject should be asked: "How was your hayfever this year compared with years before you started immunotherapy treatment (Tick only one)?

**Assessment**						
Much better (+3)	Better (+2)	A little better (+1)	The same (0)	A little worse (-1)	Worse (-2)	Much worse (-3)
