# Supplementary material for: Protocol for a double-blind randomised controlled trial of low dose intradermal grass pollen immunotherapy versus a histamine control on symptoms and medication use in adults with seasonal allergic rhinitis (PollenLITE)
Source: Clin Transl Allergy. 2013 Aug 21;3:27. doi: 10.1186/2045-7022-3-27 (PMC3765857; doi:10.1186/2045-7022-3-27)
Supplement: Additional file 3 — Procedures for Recording and Reporting Adverse Events. [file 2045-7022-3-27-S3.pdf]

ADDITIONAL INFORMATION

Procedures for Recording and Reporting Adverse Events

The Medicines for Human Use (Clinical Trials) Regulations 2004 and Amended Regulations 2006 gives the following definitions:

Adverse Event (AE): Any untoward medical occurrence in a subject to whom a medicinal product has been administered including occurrences which are not necessarily caused by or related to that product.
Adverse Reaction (AR): Any untoward and unintended response in a subject to an investigational medicinal product which is related to any dose administered to that subject.
Unexpected Adverse Reaction (UAR): An adverse reaction the nature and severity of which is not consistent with the information about the medicinal product in question set out in:

The summary of product characteristics (SmPC) for that product (for products with a marketing authorisation)
The Investigator's Brochure (IB) relating to the trial in question (for any other investigational product)

Serious Adverse Event (SAE), Serious Adverse Reaction (SAR) or Unexpected Serious Adverse Reaction (USAR): Any adverse event, adverse reaction or unexpected adverse reaction, respectively, that

Results in death;
Is life-threatening;
Required hospitalisation or prolongation of existing hospitalisation;
Results in persistent or significant disability or incapacity;
Consists of a congenital anomaly or birth defect.

Although not a serious adverse event, any unplanned pregnancy should be reported via the SAE reporting system as stated below.

Reporting Responsibilities
King's College London and Guy's and St Thomas' NHS Foundation Trust as co-sponsors have delegated the delivery of the Sponsor's responsibility for Pharmacovigilance (as defined in Regulation 5 of the Medicines for Human Use (Clinical Trials) Regulations 2004 to the King's Health Partner's Clinical Trials Office (KHPCTO).

All SAEs, SARs and SUSARs (excepting those specified in this protocol as not requiring reporting) will be reported immediately by the Chief Investigator to the KHPCTO in accordance with the current Pharmacovigilance Policy.

The KHPCTO will report SUSARs to the regulatory authorities (MHRA, competent authorities of other EEA (European Economic Area) states in which the trial is taking place.
The Chief Investigator will report to the relevant ethics committee. Reporting timelines are as follows:

- SUSARs which are fatal or life-threatening must be reported not later than 7 days after the sponsor is first aware of the reaction. Any additional relevant information must be reported within a further 8 days.
- SUSARs that are not fatal or life-threatening must be reported within 15 days of the sponsor first becoming aware of the reaction.

The Chief Investigator and KHPCTO (on behalf of the co-sponsors), will submit a Development Safety Update Report (DSUR) relating to this trial IMP, to the MHRA and REC annually.
